# The Role of microRNAs in Gene Expression and Signaling Response of Tumor Cells to an Acidic Environment

**DOI:** 10.3390/ijms242316919

**Published:** 2023-11-29

**Authors:** Anne Riemann, Mandy Rauschner, Sarah Reime, Oliver Thews

**Affiliations:** Julius Bernstein Institute of Physiology, University of Halle-Wittenberg, 06108 Halle, Germany

**Keywords:** tumor acidosis, microRNA, gene expression, cytokines, signaling pathways, MAPK, PKC, ROS

## Abstract

Many tumors are characterized by marked extracellular acidosis due to increased glycolytic metabolism, which affects gene expression and thereby tumor biological behavior. At the same time, acidosis leads to altered expression of several microRNAs (*Mir7*, *Mir183*, *Mir203*, *Mir215*). The aim of this study was to analyze whether the acidosis-induced changes in cytokines and tumor-related genes are mediated via pH-sensitive microRNAs. Therefore, the expression of *Il6*, *Nos2*, *Ccl2*, *Spp1*, *Tnf*, *Acat2*, *Aox1*, *Crem*, *Gls2*, *Per3*, *Pink1*, *Txnip*, and *Ypel3* was examined in acidosis upon simultaneous transfection with microRNA mimics or antagomirs in two tumor lines in vitro and in vivo. In addition, it was investigated whether microRNA expression in acidosis is affected via known pH-sensitive signaling pathways (MAPK, PKC, PI3K), via ROS, or via altered intracellular Ca^2+^ concentration. pH-dependent microRNAs were shown to play only a minor role in modulating gene expression. Individual genes (e.g., *Ccl2*, *Txnip*, *Ypel3*) appear to be affected by *Mir183*, *Mir203*, or *Mir215* in acidosis, but these effects are cell line-specific. When examining whether acid-dependent signaling affects microRNA expression, it was found that *Mir203* was modulated by MAPK and ROS, *Mir7* was affected by PKC, and *Mir215* was dependent on the intracellular Ca^2+^ concentration. *Mir183* could be increased by ROS scavenging. These correlations could possibly result in new therapeutic approaches for acidotic tumors.

## 1. Introduction

In addition to the pronounced oxygen deficiency in tumor tissues, extracellular acidification plays an important role as a metabolic stress factor for tumor cells and also for normal cells (e.g., fibroblasts, immune cells), which are part of the tumor tissue. On the one hand, tumor acidosis is the result of insufficient O_2_ supply to the tissue (due to inadequate tumor perfusion), but on the other hand, it may also be caused by the phenomenon of aerobic glycolysis, which has been a well-known phenomenon in tumor cells for many years. It has already been shown in various studies, that the acidic extracellular environment can lead to functional changes in tumor cells and thereby influence the malignant behavior of tumors. Thus, experimental studies have shown that a lowered pH leads to changes in cell–cell and cell–matrix adhesion [1,2,3], increases the migratory activity of tumor cells [4,5], and thereby promotes metastasis [6]. The expression of numerous proteins is affected by acidosis [3,7]. For example, epithelial–mesenchymal transition (EMT) is promoted [8], but also markers of cell proliferation and cell death are affected. It has also been shown that the activity of drug transporters responsible for the development of multidrug resistance is enhanced under acidotic conditions [9]. Finally, the secretion of inflammatory cytokines and the phagocytosis activity of monocytes and macrophages are also altered in acidosis [10].

These numerous effects of acidosis lead directly to the question of the mechanism by which extracellular pH affects gene expression and functional parameters. In studying various signaling pathways, it has already been shown that the MAP kinases p38 and ERK1/2 are activated by acidosis [11,12] and the PI3K/Akt and PKC (protein kinase C) pathways are affected [9,13]. Also, the formation of reactive O_2_ species (ROS) is stimulated by extracellular acidosis [11] and there is a decrease in intracellular Ca^2+^ concentration [9]. However, the exact mechanism by which pH triggers these changes in signaling cascades is not yet clear.

In particular, other mechanisms influencing gene expression must also be considered. For the milieu factor hypoxia, it is known that O_2_ deficiency in tumors can lead to altered expression of non-coding RNA (e.g., microRNA). For example, hypoxia leads to an increase in *Mir210*, or decreased expression of *Mir34* via the activation of HIF-1α. In light of this, it has been shown that extracellular acidosis can also lead to altered expression of various microRNAs. In a previous study the expression of numerous microRNAs was analyzed by microRNA sequencing and qPCR, and it was found that several microRNAs were affected by the low extracellular pH [14]. The most strongly regulated microRNAs were *Mir7*, *Mir96*, *Mir133b*, *Mir141*, *Mir7*, *Mir144*, *Mir183*, *Mir200c*, *Mir203*, and *Mir215*. However, validating the results in another tumor cell line led to different results. Only four microRNAs were uniformly regulated by acidosis in different tumor lines. *Mir7* was up-regulated and *Mir183*, *Mir203*, and *Mir215* were down-regulated [14]. The pH-dependent regulation of *Mir7* and *Mir203* was also confirmed in human tumor cell lines (Supplementary Figure S1). For this reason, the present study analyzed the impact of these four pH-dependent microRNAs on gene expression.

MicroRNAs are an important regulator of post-transcriptional gene regulation. MicroRNAs are short (~22-nucleotide) single-strand non-coding RNAs. They are formed in a multi-step process from pri-microRNAs which are cleaved, exported from the nucleus, integrated in a complex with DICER1, and finally leading to single-strand mature microRNAs [15]. The mature microRNA can interact with mRNA of different genes to inhibit translation. Inhibition acts by binding the microRNA sequence to the respective complementary strand of the mRNA. If the complementary is perfect the mRNA is eliminated. If the complementary is imperfect the translation is only repressed [16]. In oncology the inhibition of translation can have different effects. If the microRNA target is an oncogene, a reduced microRNA expression will lead to a pro-oncogenic effect, whereas a higher microRNA level will act antitumorigenic. If the microRNA target is a tumor suppressor the effect of an altered microRNA expression will be reversed [17]. The target genes affect every step of tumor progression or repression. For instance, *Mir203*, which was shown to be acidosis-dependent, can modulate the expression of *ABCE1* which in turn affects EMT or cancer progression [18,19].

Using bioinformatic techniques various genes can be predicted as targets of the abovementioned pH-dependent microRNAs, which could be associated with the functional changes described above. Therefore, the question arises of whether tumor acidosis could mediate the observed effects on inflammatory cytokine expression or on genes involved in proliferation or metastasis via pH-dependent microRNAs.

Against this background, the present study aimed to analyze whether the altered expression of the inflammatory genes *Il6* (interleukin 6), *Nos2* (nitric oxide synthase 2), *Ccl2* (C-C motif chemokine ligand 2), *Spp1* (secreted phosphoprotein 1), and *Tnf* (tumor necrosis factor alpha) induced by extracellular acidosis, as well as the tumor-relevant genes *Acat2* (acetyl-CoA acetyltransferase 2), *Aox1* (aldehyde oxidase 1), *Crem* (cAMP responsive element modulator), *Gls2* (glutaminase 2), *Per3* (period circadian regulator 3), *Pink1* (PTEN induced kinase 1), *Txnip* (thioredoxin interacting protein), and *Ypel3* (yippee like 3) can be regulated by the abovementioned pH-dependent microRNAs. The investigations were carried out both in vitro and in vivo (in experimental tumors). For this purpose, tumor cells were exposed to extracellular acidosis and expression was compared to control conditions. At the same time, the cells were transfected either with the microRNAs themselves (mimics) or with their inhibitors (antagomirs) to mimic or antagonize the conditions under acidosis.

The hypothesis described so far assumes that acidosis alters the expression of microRNAs and that these modulate gene expression either directly or via other signaling pathways (e.g., MAP kinases, PI3K). However, there is also evidence that signaling pathways (PKC, MAPK) themselves can affect the expression of microRNAs. Furthermore, because it is currently unclear how extracellular acidosis leads to altered expression of *Mir7*, *Mir183*, *Mir203*, and *Mir215*, we also investigated whether the previously described signaling pathways (MAP kinases, PI3K/Akt, PKC), increased ROS formation, or decreased intracellular Ca^2+^ concentration, to alter microRNA expression. To do so, cells were exposed to acidosis and incubated with inhibitors of the respective signaling pathways, and the expression of pH-sensitive microRNAs was subsequently determined.

## 2. Results

### 2.1. Genes of Inflammatory Mediators

**Figure 1:**
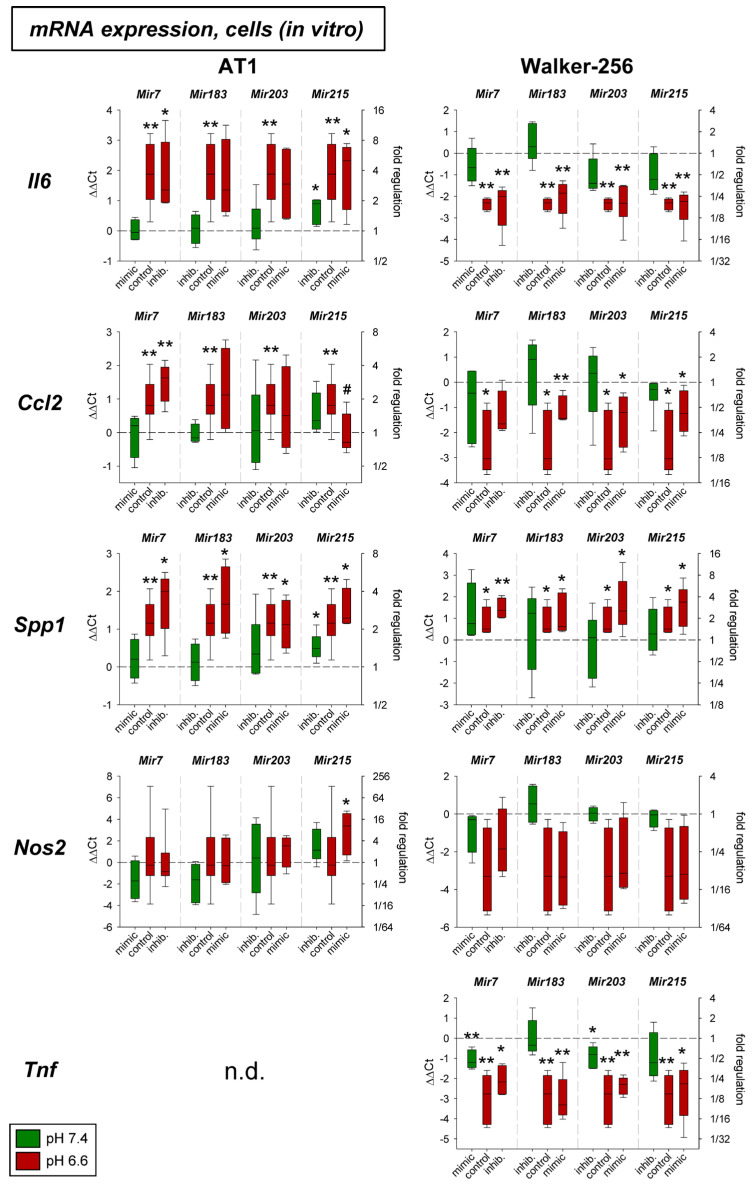
mRNA expression of inflammatory genes in AT1 and Walker-256 carcinoma cells after 24 h at pH 7.4 or pH 6.6 in combination with overexpression (mimic) or inhibition (inhib.) of pH-dependent microRNAs. n = 4–16, (*) *p* < 0.05, (**) *p* < 0.01 vs. pH 7.4 control; (#) *p* < 0.05 vs. pH 6.6 control; n.d. not detectable.

Figure 1 shows the impact of acidosis with and without modification of the microRNA expression in both tumor cell lines in vitro. Acidosis per se had a strong impact on almost all genes. Only *Nos2* (iNOS) was slightly affected by low extracellular pH. *Il6* and *Ccl2* showed a disparate behavior to acidosis in the two different tumor cell lines studied. Additional transfection with microRNA mimics or inhibitors showed mostly no effect. Only *Mir215* in AT1 cells led to changes in mRNA expression. For *Il6* and *Spp1* at normal pH the inhibition of *Mir215* led to an increased expression, and for *Ccl2* the acidosis-induced increase could be counteracted by *Mir215* overexpression reducing the *Ccl2* expression to the control level. These results may indicate that *Mir215* plays a role in regulating the expression of these inflammatory genes and that their altered expression during acidosis might—at least partially—be mediated by changes in *Mir215* expression.

**Figure 2:**
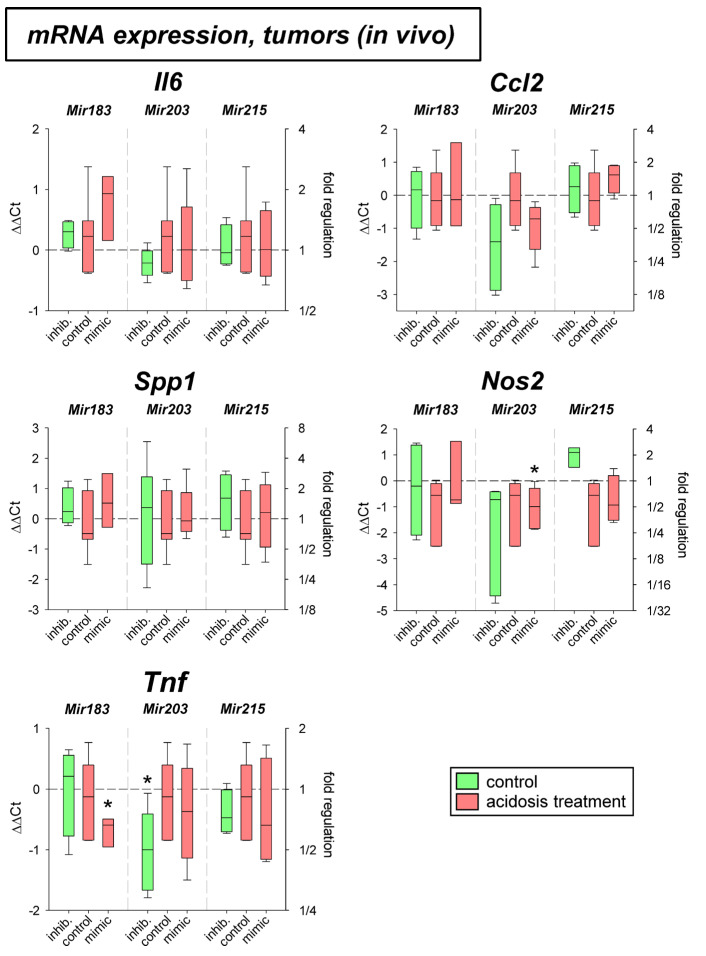
mRNA expression of inflammatory genes in AT1 tumors in vivo after 24 h under control or acidotic conditions in combination with overexpression (mimic) or inhibition (inhib.) of pH-dependent microRNAs. n = 3–7, (*) *p* < 0.05 vs. pH 7.4 control.

Figure 2 shows the results of the experiments in experimental AT1 tumors in vivo. Here neither acidosis alone nor additional overexpression or inhibition of the microRNAs had a systematic impact on the expression of the inflammatory genes analyzed.

### 2.2. Genes Related to Tumor Progression

**Figure 3:**
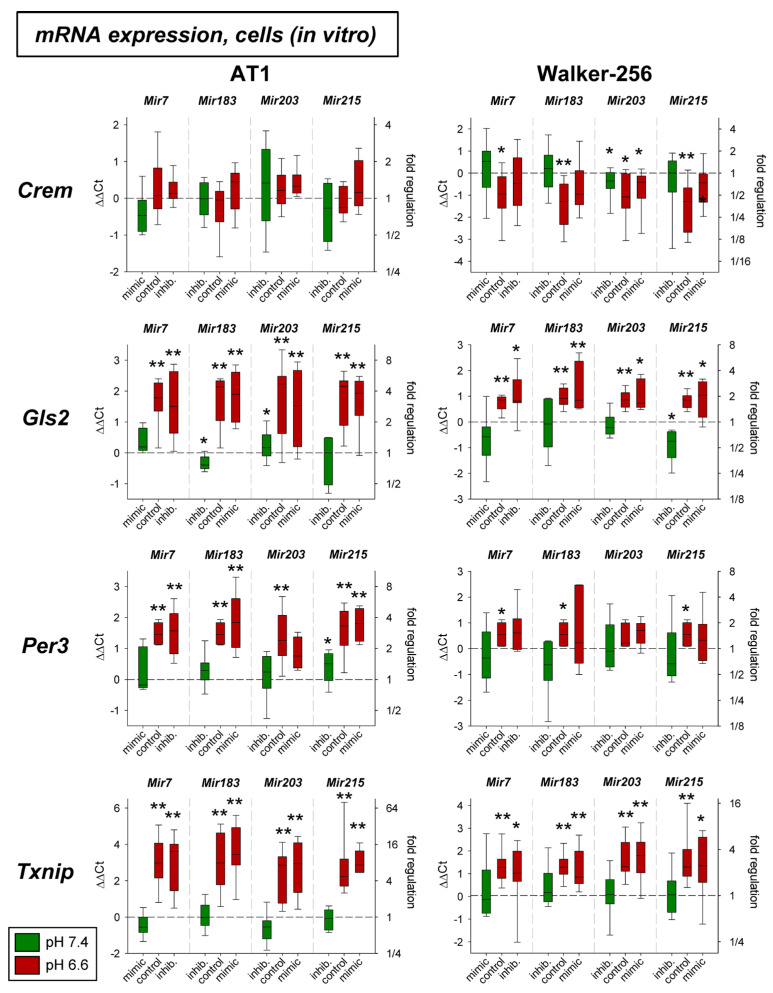
mRNA expression of tumor-associated genes in AT1 and Walker-256 carcinoma cells after 24 h at pH 7.4 or pH 6.6 in combination with overexpression (mimic) or inhibition (inhib.) of pH-dependent microRNAs. n = 4–20, (*) *p* < 0.05, (**) *p* < 0.01 vs. pH 7.4 control.

**Figure 4:**
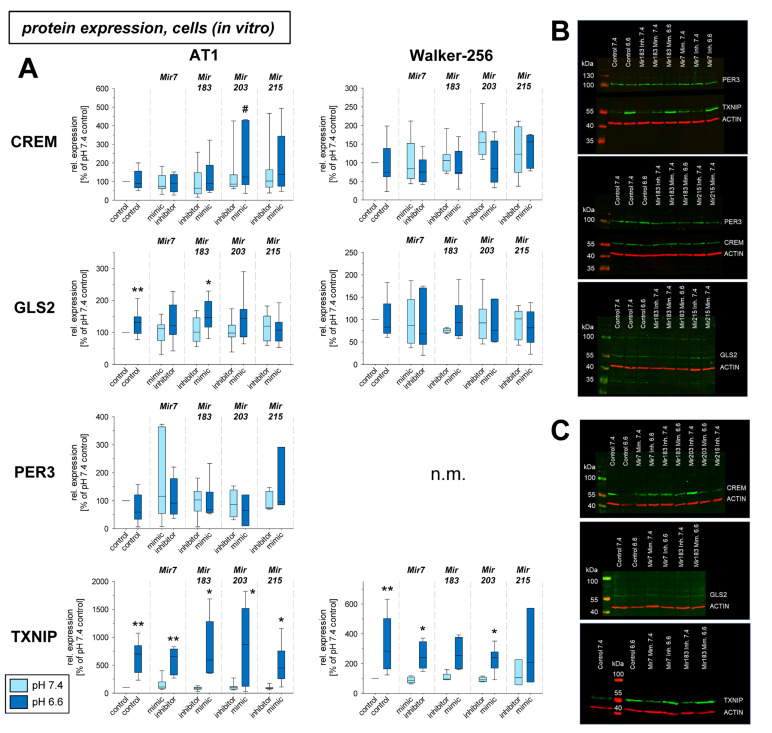
(**A**) Protein expression of tumor-associated genes in AT1 and Walker-256 carcinoma cells after 24 h at pH 7.4 or pH 6.6 in combination with overexpression (mimic) or inhibition of pH-dependent microRNAs. Example Western blots of (**B**) AT1 and (**C**) Walker-256 cells. n = 2–24, (*) *p* < 0.05, (**) *p* < 0.01 vs. pH 7.4 control; (#) *p* < 0.05 vs. pH 6.6 control; n.m. not measured.

In a previous study the mRNA expression of different genes under acidic conditions was analyzed and it was found that several genes were up- or down-regulated [3]. For eight of these genes (*Crem*, *Gls2*, *Per3*, *Txnip*, *Acat2*, *Aox1*, *Pink1*, *Ypel3*), it was tested whether for the pH-dependent regulation the pH-sensitive microRNAs are relevant. Figure 3 shows mRNA expression of *Crem*, *Gls2*, *Per3*, and *Txnip* in cells exposed to acidic pH with concomitant overexpression and inhibition of the four microRNAs analyzed. *Gls2*, *Per3*, and *Txnip* were up-regulated at low pH and *Crem* was unaffected in AT1 cells but down-regulated in Walker-256 cells. The additional transfection with microRNA mimics or inhibitors had almost no impact. For these genes the protein expression was also analyzed (Figure 4). In this group of tumor-relevant proteins, only TXNIP was significantly upregulated under acidotic conditions in both cell lines. The transfection with *Mir7*, *Mir183*, and *Mir203* had almost no impact. However, in Walker-256 cells the transfection with *Mir215* mimics under acidosis reduced the elevated TXNIP expression to the control level.

**Figure 5:**
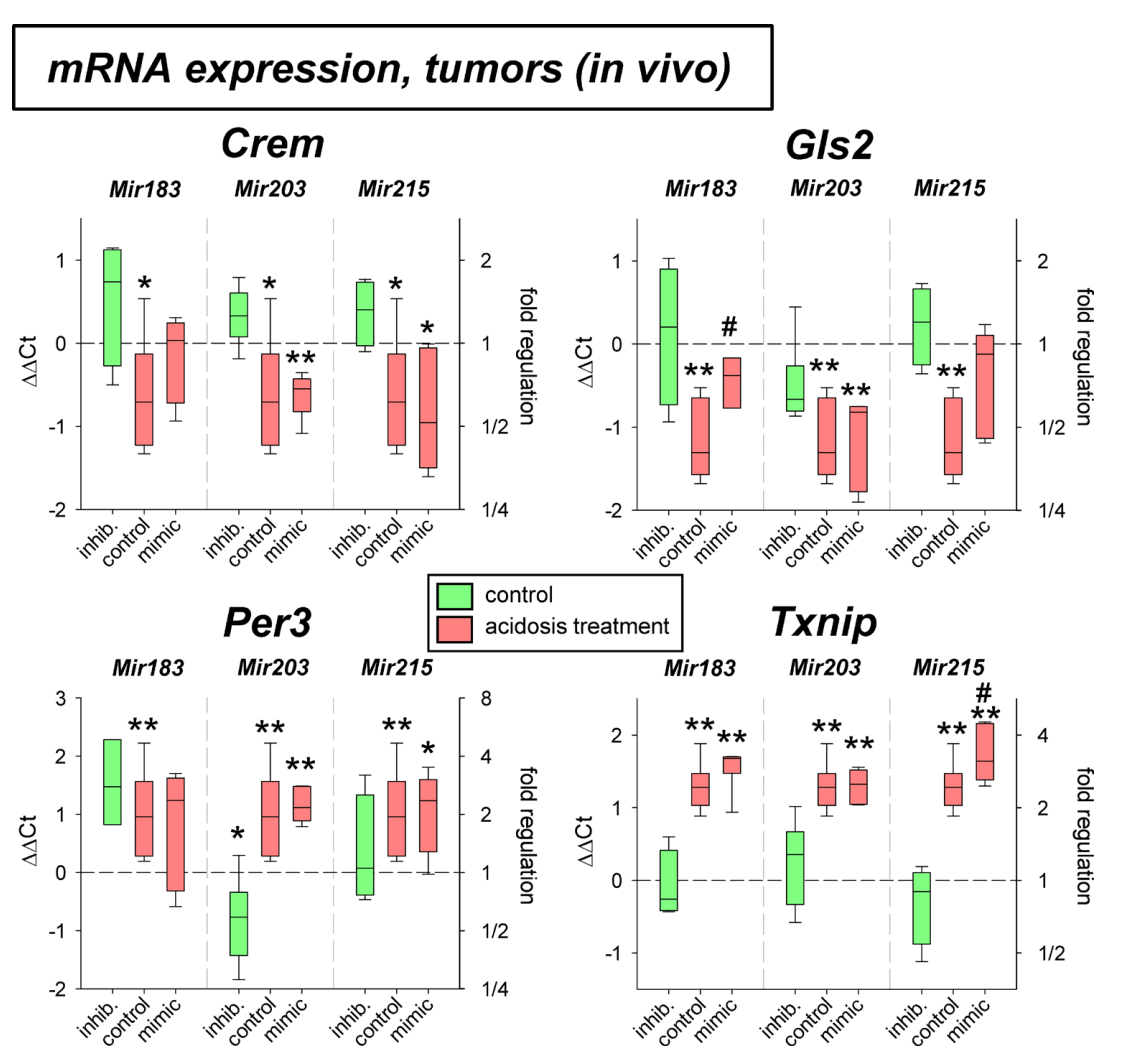
mRNA expression of tumor-associated genes in AT1 tumors in vivo after 24 h under control or acidotic conditions in combination with overexpression (mimic) or inhibition (inhib.) of pH-dependent microRNAs. n = 3–9, (*) *p* < 0.05, (**) *p* < 0.01 vs. pH 7.4 control, (#) *p* < 0.05 vs. pH 6.6 control (without transfection at the respective pH).

**Figure 6:**
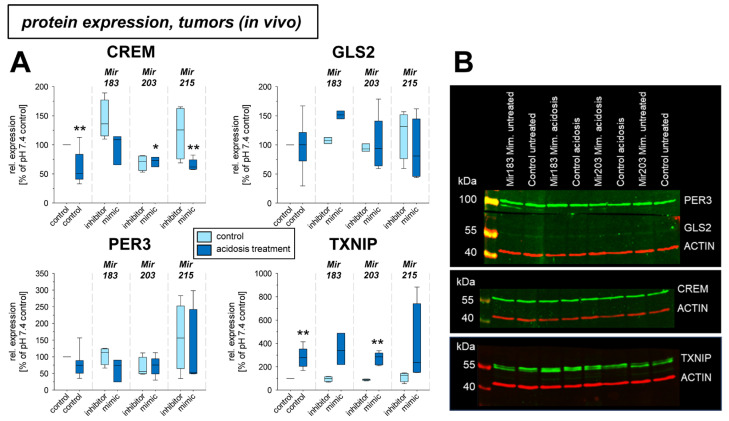
(**A**) Protein expression of tumor-associated genes in AT1 tumors in vivo after 24 h under control or acidotic conditions in combination with overexpression (mimic) or inhibition of pH-dependent microRNAs. (**B**) Example Western blots. n = 2–14, (*) *p* < 0.05, (**) *p* < 0.01 vs. pH 7.4 control.

Under in vivo conditions in experimental tumors *Per3* and *Txnip* mRNA was elevated by the acidosis treatment (Figure 5), whereas *Gls2* expression was significantly lowered. The transfection with microRNAs showed an effect, only for *Mir215* mimics at low pH. Here, the reduced *Gls2* expression was slightly elevated and the *Txnip* expression was further increased even above the level of acidotic control. The protein expression in vivo showed, however, a different picture (Figure 6). In experimental tumors the overexpression of *Mir183* under acidic conditions led to a significant increase of CREM and GLS2 protein. The overexpression of *Mir203* at low pH, however, reduced the acidosis-induced elevated TXNIP expression to the control level. Finally, overexpression of *Mir215* increased the PER3 expression during the acidosis treatment.

**Figure 7:**
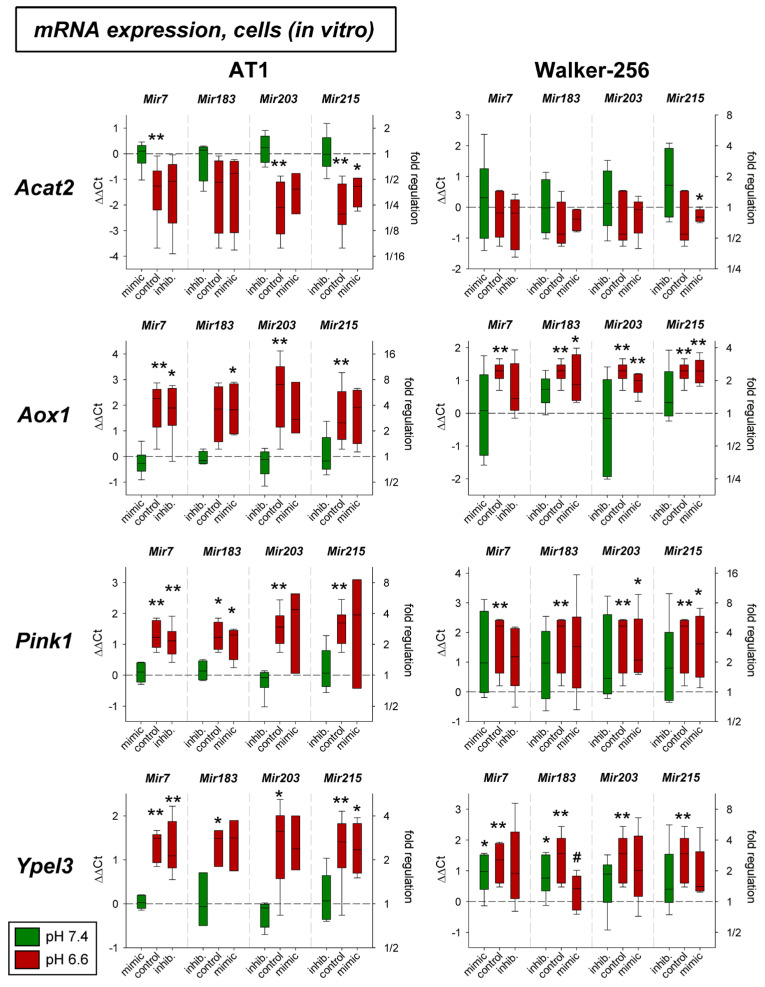
mRNA expression of tumor-associated genes in AT1 and Walker-256 carcinoma cells after 24 h at pH 7.4 or pH 6.6 in combination with overexpression (mimic) or inhibition (inhib.) of pH-dependent microRNAs. n = 3–7, (*) *p* < 0.05, (**) *p* < 0.01 vs. pH 7.4 control, (#) *p* < 0.05 vs. pH 6.6 control (without transfection at the respective pH).

In addition, the expression of four other genes (*Acat2*, *Aox1*, *Pink1*, *Ypel3*) was tested solely on the mRNA level. As shown in Figure 7 the expression of *Aox1*, *Pink1*, and *Ypel3* was up-regulated by acidosis in both cell lines, whereas *Acat2* was down-regulated, however, only in AT1 cells. The additional transfection with mimics or antagomirs of the different microRNAs had almost no impact. Only the overexpression of *Mir215* under acidic conditions led to a significant reduction of *Ypel3*.

**Figure 8:**
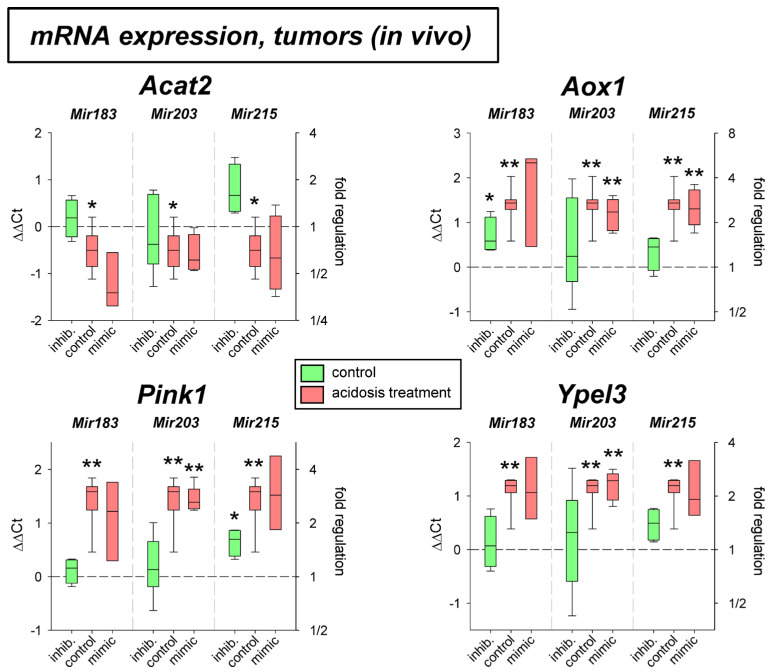
mRNA expression of tumor-associated genes in AT1 tumors in vivo after 24 h under control or acidotic conditions in combination with overexpression (mimic) or inhibition (inhib.) of pH-dependent microRNAs. n = 3–9, (*) *p* < 0.05, (**) *p* < 0.01 vs. pH 7.4 control.

The effect of acidosis alone (up-regulation of *Aox1*, *Pink1*, and *Ypel3* and down-regulation of *Acat2*) was seen in AT1 tumors in vivo (Figure 8). In these tumors, however, none of the microRNAs showed any impact on mRNA expression.

### 2.3. microRNA Expression and Signaling Pathways

**Figure 9:**
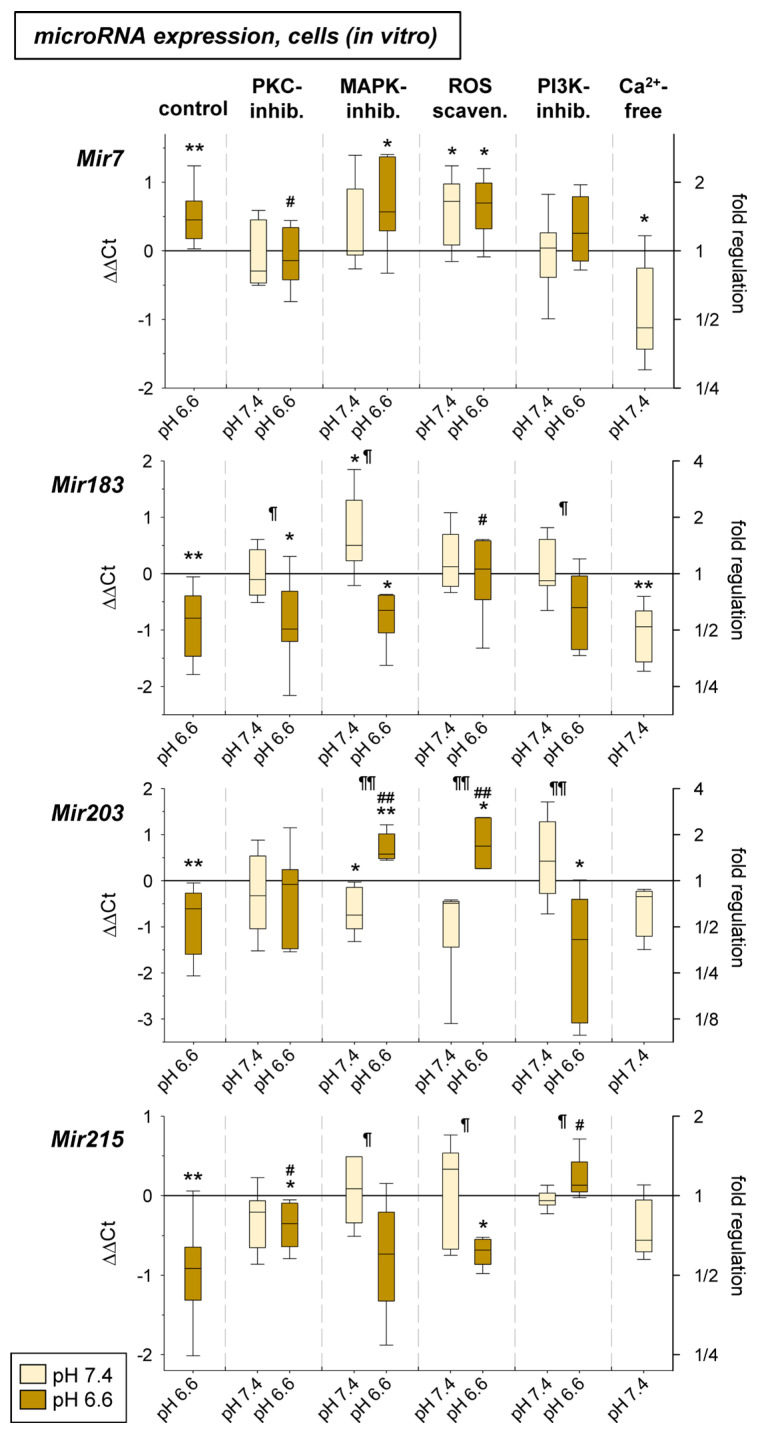
microRNA expression in AT1 carcinoma cells after 24 h at pH 7.4 or pH 6.6 in combination with an inhibition of different signaling pathways (PKC: BIM bisindolylmaleimide 1 µM; MAPK: U0126 10 μM + SB203580 10 μM; ROS: Tiron 1 mM; PI3K: Wortmannin 0.1 µM; Ca^2+^: ~5 µM). n = 4–17, (*) *p* < 0.05, (**) *p* < 0.01 vs. pH 7.4 control, (#) *p* < 0.05, (##) *p* < 0.01 vs. pH 6.6 control, (¶) *p* < 0.05, (¶¶) *p* < 0.01 pH 6.6 vs. pH 7.4 (both with inhibition).

In the third part of the study, it was tested whether signaling pathways known to be activated by acidosis [9,11] may be responsible for the acidosis-induced change in microRNA expression. Here, we analyzed PKC, MAP kinase, and Akt/mTOR signaling as well as the role of reactive oxygen species (ROS) and low intracellular Ca^2+^ (both already shown to be modulated by low extracellular pH [9,11]). To analyze the role of the signaling cascades the pathways were blocked by chemical inhibitors (PKC by BIM 1 µM; MAPK by U0126 10 μM + SB203580 10 μM; PI3K by Wortmannin 0.1 µM). ROS generation was reduced by Tiron (1 mM) and low intracellular Ca^2+^ was induced by incubating the cells in Ca^2+^-free medium (Ca^2+^: ~5 µM) [9]. With these methods the signaling pathways were blocked during extracellular acidosis and the expression of the four pH-dependent microRNAs was measured. As Figure 9 shows, inhibition of the PKC reduced the acidosis effect on *Mir7* (increased by acidosis) and *Mir215* (reduced expression). Inhibition of the MAPKs had a strong effect on the expression of the *Mir203*, which was down-regulated under acidic conditions but was highly up-regulated with the combination of low pH and MAPK inhibition. Inhibition of the PI3K/Akt pathway reversed the effect of acidosis on *Mir215* expression resulting in a strong up-regulation. ROS scavenging led to a significant increase in the expression of *Mir183* and *Mir203* under low pH conditions. Finally, the induction of low intracellular Ca^2+^ reduced the expression of the *Mir7* (opposite to an extracellular acidosis) as well as the *Mir183*, which corresponds to the effect of extracellular acidosis.

## 3. Discussion

In the present study, we investigated whether the alteration of gene expression of various inflammatory mediators and tumor-relevant proteins caused by extracellular acidosis may be mediated by pH-sensitive microRNAs. For this purpose, cells were transfected with mimics or inhibitors of the corresponding microRNAs. For *Mir7*, which is overexpressed under acidosis, mimic transfection at normal pH should mimic the acidosis situation and inhibitor transfection at low pH should antagonize the acidosis effect. For *Mir183*, *Mir203*, and *Mir215*, which are decreased in acidosis, the opposite transfection was chosen.

### 3.1. Inflammatory Mediators

In cell culture experiments, an increase in the expression of almost all mediators was observed under acidic control conditions in both cell lines (Figure 1). These results are in good agreement with previous experiments in tumor and normal cell lines [10,20,21]. On the other hand, measurements in experimental tumors in vivo could not confirm these acidosis effects. In artificially acidified tumors, there was no increase in mRNA of the mediators studied (Figure 2). Such a difference has also been observed by other authors. It was shown that different acid anions had different effects on cytokine secretion. For example, in RAW 264.7 cells acidification with HCl led to an increase in iNOS expression, whereas acidification with lactic acid led to a decrease in expression [22]. Also, the interaction of normal and tumor cells in tissue in vivo may influence expression [23].

The influence of pH-dependent microRNAs on expression showed almost no effect. Only overexpression of *Mir215* led to a significant decrease in *Ccl2* expression in AT1 cells under acidotic conditions (Figure 1). Here, transfection of the cells with the *Mir215* mimic reduced *Ccl2* expression to approximately the control level at pH 7.4. These results may suggest that *Mir215* expression has a relevant effect on *Ccl2* expression under acidosis. Although the *Ccl2* gene does not have a target region for *Mir215*, so that direct influence by microRNA is unlikely, indirect effects via other signaling pathways must also be considered. For example, *Mir215* is known to affect the PI3K pathway [24,25], which in turn can then modulate *Ccl2*. Also, the interference of *Mir215* expression with the PI3K pathway observed in the present work (Figure 9) may be indicative of functional coupling of *Mir215* with pH-dependent signaling pathways.

### 3.2. Genes Related to Tumor Progression

Concerning genes regulated by pH-dependent microRNAs the bioinformatic software miRWalk version 2.0 [26] was used to predict possible target genes. MiRWalk2.0 combines the information of possible miRNA binding sites within the complete sequence of a gene from several miRNA-target prediction databases (e.g., miRBase Targetscan, miRTarBase). The list of possible target genes of each pH-dependent miRNA was then analyzed using the Database for Annotation, Visualization and Integrated Discovery (DAVID) [27,28] to determine further possible functions of these genes. The analysis revealed that genes regulated by *Mir7* play a role for transcription control, cell morphogenesis, and nutrient metabolism. Additionally, this microRNA affects the activation of several signaling cascades (e.g., ERK1/2). These processes might affect cell proliferation [29,30,31] and several processes of malignant progression including multidrug resistance. In other studies, it was also shown that overexpression of Mir7 (as found under acidic conditions) can increase tumor cell migration [31,32] and tumor cell invasion [33], both important steps of metastasis formation. Genes regulated by *Mir183* are involved in DNA damage repair relevant for sensitivity to non-surgical treatment modalities. *Mir183* also has an impact on proliferation by regulating different genes such as low-density lipoprotein receptor-related protein 6 (LRP6) or Forkhead box O1 (FoxO1) [34,35,36]. Also, several genes relevant for cell–cell and cell–matrix adhesion are under control of this microRNA which has an impact on metastatic spread and invasiveness of tumors [37,38,39]. *Mir203* is strongly related to growth regulation and morphogenesis which could have a stimulating effect on tumor growth [40,41,42]. This microRNA affects cell adhesion (positively cell–cell adhesion, negatively cell–matrix adhesion) which could have an impact on different steps of metastasis formation [42,43,44,45]. *Mir215* seems to be involved in stress response (similar to response to hypoxia) and to autophagy. This microRNA was also demonstrated to suppress tumor proliferation [46,47,48] which is of high importance since acidosis leads to a reduced *Mir215* expression. This microRNA was also demonstrated to modulate tumor cell migration and invasiveness [49,50]. Surprisingly for three of the four pH-dependent microRNAs a regulation of genes related to biological rhythms was predicted (e.g., *Per3* period circadian regulator 3). However, the role of these biological rhythm associated genes for oncology remains currently unclear.

Analyzing the signaling pathways which are under control of the pH-dependent microRNAs shows that the mTOR pathway is regulated by *Mir7* and *Mir183*. Akt signaling was described as being affected by *Mit215* [51]. MAP kinases are modulated by *Mir183*, whereas *Mir203* has an impact on IP3 signaling and the Ras/Raf pathway. All these signaling cascades are known to affect the malignant behavior of tumors.

In the present study, in addition to the inflammatory mediators described above, eight genes were investigated for which there was evidence from previous studies that (1) their expression might be affected by extracellular acidosis [3], and that (2) they are important for tumor malignant behavior, such as migration (e.g., *Txnip*, *Per3*, *Ypel3*), proliferation (e.g., *Crem*, *Gls2*, *Per3*, *Ypel3*), adhesion (e.g., *Pink1*, *Txnip*), metastasis (e.g., *Pink1*, *Txnip*), apoptosis, and necrosis (e.g., *Aox1*, *Acat2*, *Crem*, *Per3*, *Txnip*). For four of these genes (*Crem*, *Gls2*, *Per3*, *Txnip*), expression was determined at both the mRNA and protein levels (Figure 3 and Figure 4). Of these four genes, acidosis in vitro increased mRNA expression of *Gls2*, *Per3*, and *Txnip*, but only TXNIP was also shown to increase in protein level. The marked increase in *Txnip* expression agrees with other studies, that found *Txnip* increased, in some cases 60-fold, at an extracellular pH of 6.5 [52]. *Crem* showed no mRNA or protein change in acidosis. In experimental tumors, *Per3* and *Txnip* were upregulated by acidosis, with surprisingly decreased expression of *Gls2* by acidosis. The differences between in vitro and in vivo conditions may be due to the fact, that the experimental tumors contain normal tissue cells (e.g., fibroblasts and immune cells) in addition to tumor cells, which may show an opposite response to acidic pH.

In cell culture, there was no effect on mRNA expression by the pH-dependent microRNAs studied. Only for *Crem*, expression was there found to be reduced acidosis in Walker 256 cells, which led to an increase in *Crem* expression approximately to the control level (pH 7.4) upon simultaneous transfection with *Mir183*. A similar effect of *Mir183* was also found for *Crem* expression in AT1 tumors in vivo at both the mRNA (Figure 5) and protein (Figure 6) levels. A link between *Crem* expression and *Mir183* was previously inferred from a bioinformatics analysis of human tumors [53]. However, the correlation between *Mir183* and *Txnip* expression described for neuropathic pain could not be found in the present study in tumor cells. However, at the protein level, *Mir215* was shown to affect TXNIP expression in Walker-256 cells (Figure 4). Overexpression of *Mir215* reduced *Txnip* expression, which was increased by acidosis, back to the control level. This may indicate that *Mir215* expression, which is decreased in acidosis, may be involved in the increase in the *Txnip* level. For the TXNIP protein, a clear interference by overexpression of *Mir203* was also observed, leading to a strong reduction of the TXNIP level (Figure 6).

For four other genes (*Acat2*, *Aox1*, *Pink1*, *Ypel3*), the influence of acidosis in combination with altered microRNA expression was investigated at the mRNA level. Both in cell culture and in vivo, lowering the pH resulted in altered expression. *Acat2* was decreased expressed, whereas there was an increase in expression for *Aox1*, *Pink1*, and *Ypel3* (Figure 7 and Figure 8). Decreased *Acat* expression in acidosis was also described previously for other tumor lines [52].

With regard to the influence of the microRNAs, these four genes showed only minor effects. Only for *Ypel3*, was overexpression of *Mir183* found to reduce the otherwise increased expression back to the control level (pH 7.4) upon acidosis (Figure 7). There is no evidence in the literature for a correlation between *Mir183* and *Ypel3*. Only reduced *Mir215* was found to coincide with a decrease in *Ypel3* expression [54], but no causal link was established. For *Pink1*, associations with microRNAs were described in the literature. In endothelial cells, for example, a correlation between *Mir7* and *Pink1* seems to exist [55]. A causal relationship between the *Mir203* and *Pink1* was also shown for neurons [55,56]. To what extent these are cell line-specific effects and whether these mechanisms are relevant to tumor cells must currently remain open.

### 3.3. microRNA Expression and Signaling Pathways

There is ample evidence in the literature that microRNA expression can interfere with various intracellular signaling pathways and by this modulate the biological behavior of tumors. In principle, two different directions of interference are possible: (1) microRNAs modulate the activity of signaling pathways, and (2) activation of cellular signaling affects the expression of microRNAs. Concerning the first direction, several mechanisms have been described. MicroRNAs have been found to modulate the cellular ROS formation (e.g., regulation of ROS formation by NOX4 or modulation of ROS scavenging enzymes as the manganese-dependent superoxide dismutase) [57,58]. ROS themselves can either directly affect cellular process like genomic instability or can activate other signaling cascades like p38 or ERK1/2 MAP kinases [57]. MicroRNAs have also been described to directly affect the expression of several enzymes of the MAPK pathways [59]. *Mir203*, which was found to be pH-dependent, reduces the expression of the insulin receptor substrates 1 (IRS-1) and by this affects ERK1/2 activity [60]. A similar effect was also described for the Mir145 which also targets IRS-1 and by this can affect Akt phosphorylation [61]. Other studies show that the expression of microRNAs modulate the PI3K/Akt pathway. *Mir126* was found to directly target the p85 subunit of PI3K [62] Other mechanisms by which microRNAs can modulate Akt signaling include a reduced expression of the EGF receptor (e.g., by *Mir133b*) [63] or by a reduced expression of the Akt activator PTEN [64,65]. But also, many other proteins involved in the PI3K/Akt/mTOR way are targeted by various microRNAs [66]. Finally, the JAK/STAT3 way seems to be modulated by microRNAs. Here *Mir147* was described to downregulate STAT3 maybe in combination with the long noncoding RNA (lncRNA) *MEG3* [67].

On the other hand, activation of different intracellular signaling pathways may modulated the microRNA expression. For instance, ERK1/2 activity modulates phosphorylation of TRBP (transactivation response element RNA-binding protein) which acts as a co-factor of DICER which in turn is a key complex of microRNA maturation [68]. It was also shown that the transcription of pri-microRNA is affected by MAP kinases probably by an alteration of a microRNA-gene-promotor [69]. Guo et al. [70] found that EGF receptor activation increased the expression of *Mir145* which was inhibited by blocking ERK1/2 activation. Also, p38, which is activated in an acidic tumor environment, can modulate the expression of microRNAs; however, the underlying mechanism is still unclear [71]. Akt also modulates microRNA expression. *Mir21* was shown to be upregulated whereas *Mir199a* was repressed upon Akt activation [62]. Here indication was found that the microRNAs are post-transcriptionally regulated by different members of the SMAD family. Another possible mechanism by which PI3K/Akt/mTOR signaling can affect microRNA expression could be via Mdm2-dependent ubiquitination of DROSHA, which is an essential component of the microprocessor complex for the formation of pri-microRNA [66]. DROSHA (as well as DICER) can be directly affected by reactive oxygen species and through this can modulate the expression of numerous microRNAs [58].

To analyze the role of signaling pathways on the expression of pH-dependent microRNAs we investigated whether PKC, MAP kinases, PI3K/Akt signaling or other intracellular messengers (ROS, Ca^2+^ ions), which are known to be altered by acidosis, could have caused the observed changes in microRNA expression. Inhibition of protein kinase C had an inhibitory effect on *Mir7* expression under acidotic conditions (Figure 9). In the literature, the influence of PKC on *Mir15a* and *Mir200c* has been described so far [72,73], but not for *Mir7*. Future studies should analyze the mechanism by which activation or inhibition of PKC may lead to modulation of miR expression and whether this mechanism has functional significance for tumor cells. Regarding MAP kinases p38 and ERK1/2, it was shown that upon inhibition, *Mir203* was significantly altered during acidosis. Whereas this microRNA was decreased in acidosis, there was a significant increase upon inhibition of MAPK (at lowered pH) (Figure 9). Regulation of MAP kinases by *Mir203* was described previously [74], but the reverse process has only been found for *Mir7* [69,75]. Clearly, these results need further investigation, especially regarding which of the MAP kinases is responsible for the effect. However, new therapeutic approaches could also arise from such a mechanism, as *Mir203* is involved in many functional processes of tumor cells. Regarding PI3K/Akt, it was found that *Mir215* was significantly increased in expression by inhibiting the pathway by acidosis (Figure 9). Comparable to the situation of the interaction between MAPK and *Mir203*, there are findings showing activation of the PI3K/Akt pathway by *Mir215* [24,51]. On the other hand, such influence seems to be reciprocal and the PI3K/Akt pathway can modulate the expression of microRNAs [65]. However, there are no findings so far for an interaction between PI3K/Akt and *Mir215*.

Previous studies demonstrated that extracellular acidosis leads to increased ROS formation [11]. For their part, ROS can affect the expression of various microRNAs at different levels [58]. For this reason, we investigated whether ROS scavenging during acidosis affects the formation of pH-sensitive microRNAs. Here, we found that ROS scavenging resulted in a significant increase in *Mir203* (Figure 9). In the literature, evidence for such an influence comes from data of cerebral ischemia/reperfusion experiments, in which there was a reduction of *Mir203* expression via a long-non-coding RNA [56]. In addition, it has been shown that extracellular acidosis leads to a reduction of intracellular Ca^2+^ concentration by about 30–50% [9]. For the current studies, the decrease was mimicked at normal pH by incubation with a Ca^2+^-free medium. It was found that under these conditions *Mir183* was reduced comparably to acidosis (Figure 9). For the interaction between intracellular Ca^2+^ and microRNAs, both an influence of Ca^2+^ handling by microRNAs and of microRNA expression by Ca^2+^ have been described [76,77]. However, no results are yet available for *Mir183*.

### 3.4. Role of pH-Dependent microRNAs for Malignant Behavior and Therapeutic Outcome

In the present study, the influence of acidosis-dependent microRNAs on the expression of various cytokines and genes which influence the malignant behavior of tumors was investigated. In recent years, it has been shown that the extracellular pH of tumors can influence numerous functional parameters. For example, increased hematogenous metastasis was described [5], which was linked to an increased migration capacity as well as to an altered adhesion of tumor cells under acidotic conditions [3,5]. However, proliferation behavior and tumor growth were also pH-dependent [3]. Finally, it was shown that the activity of active drug transport was increased under acidotic conditions, which led to an increase in multi-drug resistance [9,78,79]. In addition to the pH-dependent changes in various intracellular signaling cascades already described in the past, the present work postulates a possible alternative mechanism by which the metabolic microenvironment may alter functional properties of tumors. Based on the present measurements, it is possible that acidosis-induced microRNAs directly influence the expression of specific relevant genes or that microRNAs indirectly interact with intracellular signaling cascades (e.g., MAP kinases, Akt/mTOR) and thereby influence the functional properties. However, no functional measurements of proliferation, metastasis, or chemoresistance were carried out in the present study. For this reason, no conclusions can be made about the validity of the hypothesis of the functional significance of microRNAs. For this purpose, extensive studies must be carried out in vitro and in vivo in the future. This study, therefore, only serves to generate a hypothesis.

These further studies should also analyze whether the acidosis-dependent microRNAs are also influenced by other parameters of the metabolic microenvironment. It has been known for some time that, for example, *Mir210* is induced by hypoxia and that this can lead to changes in gene expression and tumor cell function [80]. However, it is interesting to note that the pH-dependent microRNAs investigated in the present study (in particular *Mir203* and *Mir215*) are also associated with hypoxia [56,81,82,83,84,85]. However, other metabolic stress factors (e.g., oxidative stress or glucose deprivation) also led to altered microRNA expression, e.g., *Mir203* [86,87]. In this respect, it could be possible that the metabolic microenvironment of tumors (hypoxia, acidosis, glucose deprivation, ROS formation, etc.) influences the malignant behavior of tumor cells via common microRNA pathways.

A more precise knowledge of the relationships between the metabolic microenvironment, microRNAs, intracellular signaling pathways, and functional changes in tumor cells could also potentially lead to new therapeutic approaches. For example, pharmacological intervention in intracellular signaling cascades could influence the effect of microRNAs on cell function or modulate the expression of specific microRNAs. It would therefore be conceivable to influence the impact of metabolic milieu factors on proliferation, metastasis, or therapy resistance by interfering with microRNA expression.

## 4. Materials and Methods

### 4.1. Cell Lines

All studies were performed with two tumor cell lines of the rat: (a) subline AT1 of the Dunning prostate carcinoma R3327 (CLS # 500121, CLS GmbH, Eppelheim, Germany), and (b) Walker-256 mammary carcinoma (ATCC # CCL-38, LGC Standards GmbH, Wesel, Germany). Both cell lines were cultured with room air containing 5% CO_2_ in RPMI medium supplemented with 10% fetal calf serum (FCS) and for Walker-256 cells additionally with 10 mM L-glutamine, 20 mM HEPES, 7.5% NaHCO_3_. For the experiments, cells were incubated under serum starvation for 24 h either in medium buffered with NaHCO_3_, 10 mM MES (morpholino-ethanesulfonic acid) and 10 mM HEPES, pH adjustment to pH 7.4 or 6.6 with 1 N HCl.

### 4.2. In Vivo Tumor Models

Solid tumors of AT-1 cells were induced in vivo in male Copenhagen rats (body weight 123–279 g), housed in the animal care facility of the University of Halle. All experiments had previously been approved by the regional animal ethics committee and were conducted in accordance with the German Law for Animal Protection and the UKCCCR Guidelines [88]. Solid tumors were induced by heterotopic injection of cell suspension (6–8 × 10^6^ cells/0.4 mL isotonic saline) subcutaneously into the dorsum of the hind foot. Tumor volumes were determined by measuring the three orthogonal diameters with a caliper and with the formula: V = d_1_ × d_2_ × d_3_ × π/6. Tumors were investigated when they reached a volume of 0.48–1.63 mL.

In order to induce a more pronounced tumor acidosis in vivo, metabolic acidosis was intensified by treating tumor-bearing animals with a combination of inspiratory hypoxia and meta-iodobenzylguanidine (MIBG) which forces glycolytic metabolism [89]. Animals received an MIBG injection (20 mg/kg b.w., i.p., dissolved in isotonic saline) and were then housed in a hypoxic atmosphere containing 10% O_2_ and 90% N_2_ for 24 h. This procedure reduced the extracellular pH in AT1 tumors from 7.02 ± 0.04 to 6.48 ± 0.08 [14].

### 4.3. miRNA Transfection

To test whether four pH-dependent miRNAs (*Mir7*, *Mir183*, *Mir203*, *Mir215*) have an impact on gene and protein expression the miRNA expression was either increased or decreased by transfecting the cells with respective mimics or inhibitors (antagomirs). For the three miRNAs which were down-regulated under acidosis (*Mir183*, *Mir203*, *Mir215*) two experimental approaches were performed: (1) miR inhibitor at pH 7.4 (to mimic the miR expression at low pH), and (2) miR mimic at pH 6.6 (to antagonize the acidosis miR effect). For the *Mir7*, which was up-regulated by acidosis, the experiments were reversed ((1) mimic at pH 7.4, and (2) inhibitor at pH 6.6).

Transient transfection was performed with Lipofectamine 2000 (Thermo Fisher Scientific, Waltham, MA, USA) in accordance with the manufacturer’s instructions. In brief, AT1 and Walker-256 cells (0.5–0.7 × 10^6^ cells/mL) were incubated with lipofectamine and the respective miRNA mimics or antagomirs (miRCURY LNA, Qiagen, Hilden, Germany) for 24 h. The list of miRNA sequences used for transfection is shown in Supplementary Table S3. Transfection with unspecific miRNA sequences served as controls (Qiagen). Mimics were used at a final concentration of 1.7 nM and antagomirs at 16.7 nM. After 24 h the lipofectamine containing medium was replaced with media with different pHs, in which the cells were incubated for another 24 h.

For in vivo experiments different transfection procedures were used. For experiments with miR mimics the cells were transfected with the respective miRNA mimic and afterwards these cells were implanted as described above by subcutaneous injection. For inhibitor experiments untransfected tumor cells were implanted. When the tumors reached the desired volume a small amount (20 µL) of lipofectamine + respective miRNA inhibitor was injected intratumorally (under isoflurane anesthesia). The RNA strands are taken up into the cells and modulate the miRNA expression accordingly for at least 48 h.

### 4.4. mRNA Expression

For mRNA expression analyses, total RNA was isolated from cells or tumor homogenates using TRIzol according to the manufacturer’s instructions. For qPCR measurements 1 µg RNA was subjected to reverse transcription with SuperScript II reverse transcriptase (Thermo Fisher Scientific) and analyzed by qPCR using the Platinum SYBR Green qPCR Supermix (Thermo Fisher Scientific). The obtained data were normalized against 18S or Hprt1 and were related to the respective control. Supplementary Table S1 shows the primers used.

### 4.5. miRNA Expression

MiRNA expression in cells was assessed by Taqman-qPCR which was performed according to the manufacturer’s instructions (Taqman MicroRNA assay; Applied Biosystems, Waltham, MA, USA), normalized to unspecific snoRNA (RNU64702) or U6 snRNA, respectively, and Ct values were related to controls at pH 7.4. The list of oligonucleotides used for miRNA-PCR is shown in the Supplementary Table S2.

### 4.6. Western Blot

Western blotting was performed according to standard protocols. In brief, cells were lysed (0.5 M Tris-HCl pH 6.8; 10% SDS; 10% 2-mercaptoethanol; 20% glycerol; 0.01% bromophenol blue), separated by sodium dodecyl sulfate polyacrylamide gel electrophoresis, and transferred to a nitrocellulose membrane. Subsequently, membranes were incubated with antibodies specific for *CREM* (#PA5-29927, Invitrogen, Darmstadt, Germany), *GLS2* (#PA5-78475, Invitrogen), *PER3* (#PA5-40922, Invitrogen), and *TXNIP* (#14715, Cell Signaling, Danvers MA, USA). The bound primary antibody was visualized by IRDye secondary antibodies (Licor Biosciences, Lincoln, NE, USA) with the imaging system Odyssey (Licor Biosciences, Lincoln, NE, USA). Quantitative analysis was performed with Image Studio Lite Ver. 5.2.5 (Licor Biosciences, Lincoln, NE, USA).

### 4.7. Statistical Analysis

Results are expressed as box plots with median, 5%, 25%, 75%, and 95% percentile. Differences between groups were assessed by the two-tailed t-test for paired and unpaired samples. The significance level was set at α = 5% for all comparisons without Bonferroni correction.

## 5. Conclusions

In summary, the current study demonstrates that numerous tumor-related genes and cytokines are altered in their expression by extracellular acidosis in tumor cells both in vitro and in vivo. However, acidosis-dependent microRNAs appear to play only a minor role in this modulation. The data suggest that individual genes are affected by overexpression or inhibition of microRNAs, but these effects were cell line-specific. Nevertheless, the results raise broader questions when indirect effects (e.g., microRNA-induced changes in various signaling pathways) are included. In this context, the results of the last part of the study are of interest, in which it was shown that acidosis-activated signaling pathways (e.g., MAP kinases) can in turn influence microRNA expression. This could possibly also lead to new therapeutic approaches to influence the biological behavior of tumor cells in an acidic environment.

## Data Availability

All data generated or analyzed during this study are included in this published article and its Supplementary Materials file. The datasets used and/or analyzed during the current study are available from the corresponding author on reasonable request.

## Supplementary Materials

The supporting information can be downloaded at: https://www.mdpi.com/article/10.3390/ijms242316919/s1.
