# Supplementary material for: The Role of microRNAs in Gene Expression and Signaling Response of Tumor Cells to an Acidic Environment
Source: Int J Mol Sci. 2023 Nov 29;24(23):16919. doi: 10.3390/ijms242316919 (PMC10707721; doi:10.3390/ijms242316919)
Supplement: Supplementary file 1 [file ijms-24-16919-s001.zip › ijms-2699035-supplementary.pdf]

The role of microRNAs in gene expression and signaling response of tumor cells to an acidic environment

Anne Riemann, Mandy Rauschner, Sarah Reime, Oliver Thews
Julius Bernstein Institute of Physiology, University of Halle-Wittenberg, Germany

Supplementary Material

Table S1: Primers used for quantitative PCR.

Target	forward primer	reverse primer
Acat2	GACTGGAACGGTCACCCCAG	TCGGTCCTACTCCCATGACA
Aox1	GAGCAGACATCTGGACTCGG	GTCAGGACACTTGCGGAGAA
Crem	TCCGAGCTCCTACTACTGCT	TTTCATCAGCCTCAGCTCCC
Gls2	CCTTTTCCTTTAGATGTGGGGC	AAGCAGGTCACCAAGTCGAG
Il6	AGCCAGAGTCATTCAGAGCA	AGCATTGGAAGTTGGGGTAGG
Nos2 (iNos)	GCAGGTTGAGGATTACTTCTTCCA	GCCCTTTTTTGCTCCATAGGAAA
Ccl2 (MCP-1)	CCCAGAAACCAGCCAACT	TGCTGCTGGTGATTCTCTTG
Per3	GAACAGGAAACAACCGCACC	CCACGGGCTTGAATCCTTCT
Pink1	TGTATGAAGCCACCATGCCC	GCTTAAGATGGCTTCGCTGG
Spp1 (osteopontin)	CCTCTGAAGAAACGGATGACT	CTGGGCAACTGGGATGACCTT
Tnfa	CCACCACGCTCTTCTGTCTACTGAACT	CCATTGGCCAGGAGGGCGTT
Txnip	CCAGCCTACAGGTGAGAACG	GGCTGGGACGATCGAGAAAA
Ypel3	GCGTGCCTACCTCTTCAACT	TTGCAGTTCTCGCAGTGGAT

**Housekeeper**		
Hprt1	ACCAGTCAACGGGGGACATA	TTGGGGCTGTACTGCTTGAC
18S	CTGAGAAACGGCTACCACATC	CCCAAGATCCAACTACGAGC

Table S2: List of oligonucleotides used for miRNA-PCR.

miRNA	ID	Accession number (miRBase)	Sequence
control	snoRNA (rat)	U64702 (NCBI)	
control	U6 snRNA	NR_004394	
miR-7a-5p	rno-miR-7a-5p	MIMAT0000606	UGGAAGACUAGUGAUUUUGUUGU
miR-183-5p	rno-miR-183-5p	MIMAT0000860	UAUGGCACUGGUAGAAUUCACU
miR-203a-3p	rno-miR-203a-3p	MIMAT0000876	GUGAAAUGUUUAGGACCACUAG
miR-215	rno-miR-215	MIMAT0003118	AUGACCUAUGAUUUGACAGACA

Table S3: miRNA sequences of mimics and antagomirs used for transient transfection.

**target miRNA**	**function**	**miRNA strand**
rno-miR-7-5p	mimic	5'-UGGAAGACUAGUGAUUUUGUUGU-3'
rno-miR-7-5p	inhibitor (antagomir)	5'-ACAACAAAATCACTAGTCTTCC-3'
rno-miR-183-5p	mimic	5'-UAUGGCACUGGUAGAAUUCACU-3'
rno-miR-183-5p	inhibitor (antagomir)	5'-AGTGAATTCTACCAGTGCCAT-3'
rno-miR-203a	mimic	5'- GUGAAAUGUUUAGGACCACUAG-3'
rno-miR-203a-3p	inhibitor (antagomir)	5'-TAGTGGTCCTAAACATTTCA-3'
rno-miR-215	mimic	5'- AUGACCUAUGAAUUGACAGAC-3'
rno-miR-215	inhibitor (antagomir)	5'-GTCTGTCAATTCATAGGTCAT-3'

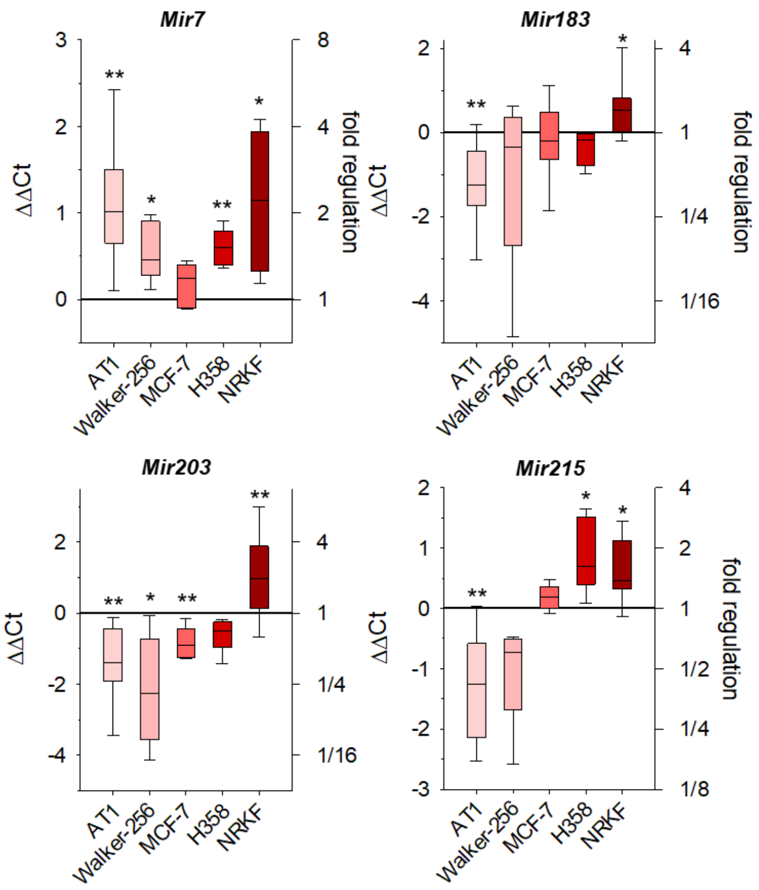

Figure S1: Expression of miRNAs under acidic conditions (pH 6.6, 24 h) in 4 tumor cell lines (AT1: subline of the rat Dunning prostate carcinoma R3327, Walker-256: rat mammary carcinoma, MCF-7: human mammary carcinoma, H358: human non-small cell lung cancer) and one normal cell line (NRKF: normal rat kidney fibroblasts). n=4-16; (*) $p<0.05$, (**) $p<0.01$ *vs.* pH 7.4.
